# Provisional Hemostasis in Operations upon the Head and Neck1Read by invitation before the Buffalo Academy of Medicine, March 3, 1903.

**Published:** 1903-06

**Authors:** George Ryerson Fowler

**Affiliations:** Brooklyn, New York. 301 DeKalb Avenue


### BUFFALO MEDICAL JOURNAL.

VOL. XLII.—LVIII. JUNE, 1903. No. 11.

### ORIGINAL COMMUNICATIONS.

### Provisional Hemostasis in Operations Upon the Head and Neck.[1]

By GEORGE RYERSON FOWLER, M.D., Brooklyn, New York.

METHODS for securing provisional or prophylactic hemostasis in general may be divided into the following: (1) elevation of the parts; (2) direct pressure upon the source of arterial supply, either manual or by the application of the tourniquet; (3) elastic constriction; (4) permanent ligation of arteries in their continuity; (5) temporary ligation of main arterial trunks; (6) provisional arrest of the blood supply after exposure of the vessel, either by means of a special clamp, a traction loop, or a twisted tape or ligature.

In operations in the region of the head, the last four find an application to a greater or lesser extent. Elastic constriction, for example, is limited to operations upon the scalp; it is most efficient in this regard, however. For instance, in the removal of large angiomatous tumors in this region, radical operative attacks upon which are attended with considerable danger to life from excessive loss of blood, removal may be effected with the minimum amount of hemorrhage by the previous application of a constricting band or cord applied on a level with the occipital protuberance behind, and the upper margin of the eyebrows in front. Rubber tubing answers the purpose excellently. To prevent slipping during the operation four stout needles may be passed through the skin upon either side of the rubber tubing at points equidistant, and stout silk thread wound figure-of-eight fashion beneath the projecting ends of the needles and over the

1. Read by invitation before the Buffalo Academy of Medicine, March 3, 1903.

constriction, as in the application of the figure-of-eight harelip suture.

Provisional permanent ligature of arteries as an immediate pre-operative measure was brought forward by Pirogoff, in 1840, and again by Madelung, in 1874. It finds its applicability in the region of the head and neck, more particularly in operative attacks upon the tongue, in which the lingual arteries are ligated, in excisions of the superior maxillæ, in which the external carotids are secured, and occasionally in thyroidectomy. In 1894, I introduced preliminary permanent ligation of the external carotid for the purpose of controlling the hemorrhage in a case of intracranial neurectomy.[1] In this case the dangers arising from loss of blood were averted, and much time saved in the operation, although there was some oozing as the dura was lifted from the base. The point selected for the application of the ligature was between the occipital and the internal maxillary, in order to cut off the blood supply from the trunk of the internal maxillary with its middle meningeal branch, and to likewise prevent hemorrhage arising from the free anastomosis of the lingual, facial and occipital branches with the corresponding vessels of the opposite side. In this case the middle meningeal artery was injured in turning down the osteo-plastic flap, made in gaining access to the cranium. It was found, however, that almost no blood was furnished by this vessel. This was in striking contrast with an experience in a previous case, in which I am convinced that the patient's death was the direct result of the blood lost during this stage of the operation.

Preliminary ligature and excision of both external carotids precedent to radical operative interference in the area of these vessels has heretofore remained an unrecognised procedure in surgical practice. Nor is it likely to be employed save under the most exceptional circumstances. Even its justifiability must remain for the present subjudice. The operation of excision of the external carotids and their branches, as introduced by Dawbarn, has found its applicability exclusively in the attempts to "starve out" otherwise supposedly inoperable malignant neoplasms situated within the area supplied by these vessels and their branches. Yet it may happen in this class of cases, that a radical operation is subsequently indicated. This is exemplified by a case occurring in my service at the German Hospital, in Brooklyn, in a patient, suffering from carcinoma of the cheek

1. New York Medical Record, June 16, 1894, p. 746.

and upper jaw. When first admitted to the hospital his general condition was so unpromising, and the local condition so far advanced, that I despaired of being able to accomplish much for his relief, and contented myself with excising both external carotids, after Dawbarn's method.

A few weeks stay in the hospital following the operation, with proper food, greatly improved his general condition. The local disease likewise showed signs of apparent improvement. The area of what appeared to be carcinomatous infiltration upon the cheek lessened very materially, and the ulcerative process ceased to be active. It is to be presumed that what was supposed to be at first an extensive invasion of the cheek was largely due to edema. At all events, the temptation was strong, in view of both the general and local improvement in the condition of the patient, to embrace the reasonable hope of an assured cure, rather than await the final results of the Dawbarn operation. So strongly did I become impressed with this as I watched him from day to day, that, after weighing carefully the chances of failure of the reparative process incident to cutting off the circulation so completely with its consequent nutritive disturbances, I finally decided to remove the upper jaw and the involved soft parts. This was done four weeks following the first operation. The operation was as bloodless as it very well could have been upon the living subject. No vessels required the application of a ligature, yet there was a sufficient amount of oozing from the incised parts to encourage the belief that repair would take place. The resulting gap in the cheek, at first not less than two and a half inches in diameter, was reduced by sliding flaps to about half that size.

The after history of the case was entirely uneventful so far as concerns the healing process. The incisions made for the sliding flaps closed by primary union, and granulating surfaces, followed by a covering of cicatricial formation, completed the repair of the raw surfaces left by the mucous membrane and bone sections. The further care of the case resolved itself into plastic replacement of the removed soft parts of the cheek, which was first attempted by a flap taken from the arm as in the old Taglicotian operation of rhinoplasty, the skin surfaces of the flap being turned in so as to prevent undue shrinkage, and at the same time serve as a substitute for the mucous membrane lining of the cheek. This was done four weeks after the original operation, delay of at least this length of time being deemed essential in order to obtain a sufficient blood supply from the edges of the defect, to insure union of these with the transplanted flap.

It is of interest in connection with our subject to discuss the final fate of the flap in this the first attempt at repair. Unfortunately, the flap itself failed to retain sufficient vitality to accomplish a result. At least two-thirds of the flap sloughed, only a comparatively narrow portion near the pedicle surviving. Wherever the flap itself did not break down, however, its edges became united to those of the defect, thus demonstrating, as had been already shown by the readiness with which the parts healed after the radical operation, that the previous excision of both external carotids need not necessarily contraindicate subsequent removal of the diseased parts, and may even have some advantages, such for instance, as holding the disease in check pending the building up of the patient, to say nothing of the complete and absolute control of the circulation in the parts for the purposes of the radical operation.[1] It is true that ligation of both external carotids will answer the last named purpose reasonably well, and therefore it is probable that, if excision of both external carotids as a preoperative measure finds a place in surgical practice at all, it will be in the class of cases of which the one herewith related is an example—namely, cases of malignant disease in this region in which the patient's general condition will not permit of operation with a reasonable assurance of success, and yet in which delay is fraught with grave danger of placing the case among the positively inoperable ones.

Temporary occlusion of the common carotids, constriction being applied sufficiently tight to occlude the vessel, and yet not injure the intima, has been in use for a number of years. I am sure that many surgeons, like myself, have taken the precaution to slip a loose ligature around the common carotid when this vessel had been laid bare in the course of a deep and extensive dissection of the neck. From this it was but a step further to make traction upon the ligature so placed to temporarily occlude the vessel and thus permit the dissection to go on unhampered by flow of blood. Such at least has been my practice for the past eighteen years.

It was not, however, until Schonborn called attention to the matter before the International Medical Congress in Rome by

---

1. This patient died of pneumonia before the work of plastic replacement of the cheek was completed.

the exhibition of an instrument designed to compress the carotids, that interest in the subject was awakened. Schonborn's contribution was followed in 1895 by the practical work of Senger, of Krefeld.[1] This in time was followed by Riese's report of two cases of resection of the upper jaw with temporary occlusion of the carotids.[2] To Dr. George W. Crile[3], of Cleveland, however, should be accorded the credit of having, in a convincing and practical manner, called renewed attention to the practicability of temporarily closing the carotid arteries for the purpose of controlling hemorrhage in certain operations upon the head and neck.

In carrying out the procedure of provisional occlusion of the carotids, thereby arresting the greater portion of the blood supply to the head, three considerations impel the surgeon to carefully weigh the measure before resorting to it. These relate, first, to the effects upon the important organs contained in the cranial cavity of depriving these of their normal blood supply for the length of time required for the operation; second, to the possibilities of the formation of coagula and the subsequent passage of these into the cerebral vessels by the restored blood stream; third, to the injurious effects of the pressure upon the arterial coats. The first two of these are of especial importance in connection with the immediate effects of "turning off" the blood stream, as it has been called. The last does not involve immediate danger to life, nor are its effects, either immediate or remote, greater when applied in this region, than when applied elsewhere.

The effects of the diminished blood supply upon the intracranial organs may be said to be practically nil, so far as our present knowledge goes. In Crile's studies, which embraced its effects upon the circulation and respiration, it was found that the blood pressure was raised at first, but the normal was soon restored by physiologic compensation. The respiratory action was decreased as the most constant of the phenomena observed, and yet this was not marked in any of the animals experimented upon. In fact, as remarked by Crile, "in no instance was there any striking results noted." It would, therefore, seem that the vertebrals are amply sufficient to carry on the circulation in the intracranial organs, so far as any knowledge of untoward effects

1. Deutsche Med. Wochenschrift, 1895, No. 22, p. 160.
2. Die Thrombose nach Versuchen und Leichenbefunden. Stuttgart, 1888.
3. Problems Relating to Surgical Operations. Philadelphia, 1901.

derived from animal experimentation, or from observations upon patients under an anesthetic, can throw light upon the subject.

It is true that compression of the carotids has been employed for therapeutic purposes in past years, and the occurrence of such symptoms as dimness of vision, vertigo, staggering gait, loss of consciousness, and even apoplectic seizures, has been attributed to the compression. Other observers have added to these dilatation of the pupils and convulsions. The want of uniformity in the results obtained in these bloodless attempts to compress the carotids on the one hand, and the absence of all untoward effects following definite and direct occlusion as exercised after exposure and isolation of the vessel, upon the other, lead to the conclusion that the symptoms attributed to compression of the carotids were really due to other causes, such as pressure upon the vagi, the jugular vein, or even upon the cervical sympathetic. The view that the effects produced were due to compression of the sensitive nerves prevailed in the age of Rufus, of Ephesus, one of the few anatomists, according to Park, mentioned by Galen. This view was also held by Galen himself.

In commenting upon the absence of marked decrease in the respiratory action in Crile's experiments, the details of the first of the eleven cases of the application of the method to the human subject should be taken into account. In this the respiration suddenly failed and artificial respiration was resorted to and continued for twenty-five minutes. The case was one, however, in which a large fibrosarcoma filled the mouth, and in which breathing was obstructed to the extent of threatening suffocation. A preliminary tracheotomy under cocain had been performed in the case. The respiratory failure took place ten minutes after the completion of the operation. The wound had been dressed and the entire cavity of the mouth packed with iodoform gauze. The patient finally recovered. Crile does not attempt to explain the failure of the respiration in this case. There does not seem to have been any obstruction in the tracheotomy tube, nor is there any statement made as to the condition of the circulation at the time of the respiratory failure.

As to the possibilities of the formation of coagulæ, and the passage of these into the cerebral vessels through the restored blood stream, it may be said that the occurrence of such an accident will depend entirely upon the care exercised in producing the occlusion. It was formerly supposed that the mere arrest of the blood at the point of obstruction was sufficient to cause the coagulation. It has been shown, however, by Alexander Schmidt, that coagulation under these circumstances is due to a ferment having its origin in the so-called blood plaques. The disintegration of the blood plaques is initiated by injury to the coats of the vessel; this disintegration, in its turn, sets free the fibrin ferment, following which coagulation of the blood at the site of the injury takes place. It would therefore follow that, if injury to the vessel walls can be avoided the dangers from this source may be escaped. The problem therefore resolves itself into some means whereby the pressure of the occluding instrument can be so regulated as to arrest the blood stream without making sufficient pressure upon the vessel to injure its walls. For this purpose Crile uses a special clamp, the jaws of which are approximated by a screw-device. When the vessel is closed the jaws of the instrument are parallel to each other. The effect of the contact of the metal with the vessel is modified by pieces of rubber tubing drawn over the jaws. This device of Crile's no doubt answers the purpose well, the adjustable set screw giving perfect control of the lumen of the vessel. He employed it in eleven cases, in all of which the instrument seems to have given entire satisfaction.

As to the third consideration—namely, that of permanent injury to the vessel itself, but very little need be said. The necessity of avoiding such injury is paramount from the fact that the immediate result of this would be the occurrence of coagulation, followed by embolic plugging of the cerebral vessels and resulting suspension of function of important intracranial organs, or even irreparable damage to the latter.

My own experience with temporary carotid occlusion is limited to two cases, one a case of gunshot wound of the neck, and the other an intracranial neurectomy. In the first case secondary hemorrhage took place on the sixth day, and was held in control by my house-surgeon at the German Hospital until my arrival, by firmly packing the wound track. The missile had entered from the direction of the cavity of the mouth, and septic conditions were present almost from the start. Without removing the packing from the wound tract, I exposed the common carotid above the omohyoid and passed two ligatures around the vessel, one of which was carefully twisted until pulsation above the ligature ceased; the other was simply clamped and held in readiness to serve as a permanent ligature should this be required in an emergency. The track of the bullet in the neck was then exposed and the blood stream turned on by untwisting the ligature. The source of the hemorrhage was then identified as the facial artery, which was easily secured. The occlusion of the common carotid was then discontinued and the wound made for its exposure sutured.

Six days later another hemorrhage took place. The carotid was again exposed by reopening the original wound, and temporarily occluded at the same point. The bleeding this time came from the direction of the inward distribution of the ascending pharyngeal branch of the external carotid. Repeated attempts to secure the vessel at the site of the bleeding proved futile. As the septic condition of the tissue in the neighborhood of the origin of the external carotid rendered the attempt to apply a ligature to the latter unsafe, I therefore proceeded to ligate the common carotid at once. In view of the possibilities of further hemorrhage through anastomosing vessels from the external carotid of the opposite side, the latter was also ligated. No disturbances from the provisional occlusion of the common carotid upon the occasion of the first operation occurred, and the patient finally made a complete recovery.

The second case occurred at the Methodist Episcopal Hospital. The patient, a man of 56, had suffered for many years from neuralgia of the 3d division of the fifth nerve of the left side. Two years ago I resected the nerve external to the cranial cavity at the foramen ovale, turning down a flap including the zygoma for the purpose. Upon his re-admission to the hospital he complained of pain in the distribution of the 2d division of the fifth nerve. His vessels were markedly atheromatous, and he presented the appearance of a much older man than his years would suggest.

On February 3, of the present year, I performed intracranial neurectomy after Abbe's method, first exposing each common carotid artery in turn and occluding it by passing a ligature about it and twisting the ends until the resulting loop constricted the vessel sufficiently to arrest pulsation. The main operation was then proceeded with, and proved the most satisfactory of its class that I have ever performed. Bleeding there was practically none, and because of this the Gasserian ganglion was quickly identified and the intracranial portions of both the 2d and 3d divisions divided close to the foramina and removed.

After placing a piece of rubber tissue over the foramina, which could be plainly discerned, the spheno-temporal lobe was replaced, a strip of iodoform gauze led from the opening of the skull to the lower angle of the wound, and the remainder of the latter closed. The provisional occlusion was then removed and bleeding watched for. None occurred.

During the operation the brain was held in a direction directly upward by an elevator with one of the miniature "cold" electric light bulbs furnished by the Surgical Appliance Manufacturing Co., of Rochester, attached. Great assistance was afforded by this instrument. During the operation nothing noteworthy occurred. The anesthetic used was chloroform. The respirations continued even and normal throughout. The pupils did not undergo any especial changes, and the heart's action was not disturbed in the least at any time.

The after history of this case is of interest as bearing upon the method of producing temporary occlusion of the carotids. Upon the day following the operation it was discovered that the patient had developed sensory aphasia. He was at a loss to employ certain words in replying to questions, but when these were supplied to him he would make use of them in their proper relation to the remainder of the sentence. This condition gradually improved, but had not disappeared altogether when he left the hospital twenty-four days after the operation. At this time the aphasia was principally manifest when he attempted to write a letter to his wife. In all other respects he appeared to be in perfect health.

The occurrence of aphasia is, of course, the point of especial interest in this case. That some lesion occurred in the pars opercularis, or region of Broca during the operation is very evident, but whether from a dislodged thrombus, which, when the force of the blood stream was released was driven into the branch of the middle cerebral, which supplies the anterior lobe, or from some injury inflicted by the elevator in lifting the central mass is not entirely clear. The patient's vessels were in a markedly atheromatous condition, and for this reason I was especially careful in applying the constriction, for I felt that, with excessive brittleness of the vessel walls, a pressure more than sufficient to arrest the pulsation in the vessel beyond the constriction might easily so disturb the integrity of these as to lead to the formation of a thrombus. It is probably true that occlusion brought about by circular constriction is more likely to inflict injury upon an atheromatous vessel than when simple flat pressure is employed, and in the future I would use a flat clamp with set-screw adjustment after the manner of Crile, in such a case. As to the pressure applied to lift the cerebral mass from the base, I can only say that this was no greater than I have employed previously upon a number of occasions, and no such accident occurred. In any event I do not feel that I shall be deterred from employing the method in future cases by this occurrence; on the other hand, I am quite convinced that too much stress cannot be laid upon the importance of exercising every care to avoid injury to the vessel.

Through the kindness of Prof. Roswell Park, I was permitted to perform intracranial neurectomy for the relief of intractable facial neuralgia before the class at the Buffalo General Hospital upon the morning of the day upon which this paper was read.

Provisional hemostasis was secured by temporary occlusion of both common carotids. There was considerable venous oozing as the cerebral mass was lifted from the base, and particularly as the 2d and 3d divisions of the fifth nerve were isolated at the foramina and divided.

In the discussion which followed the reading of this paper, Prof. Park called attention to this circumstance and stated that the explanation suggested by his service assistant, Dr. McGuire, was probably the true one—namely, that the internal jugular was more or less pressed upon by a small compress that was placed in the bight of the constricting tape to serve as a cushion to prevent injury to the artery as the former was twisted. This pressure was somewhat increased by the weight of the forceps which were clamped upon the tapes to prevent untwisting, and which were allowed to hang down and thus drag the little cushions of gauze in the direction of the vein. The remedy for this is to employ a special clamp, such as Crile recommends, for occluding the vessel. In the absence of such a special device interference with the return circulation, which is certainly a most important consideration, may be prevented by passing the clamp which secures the constricting band or ligature upon the right side across in front to the left side, and the one from the left to the right side. In this manner the constricting ligature with its cushion, if the latter be employed, together with the carotid itself, will be drawn away from the vein, and pressure upon the latter avoided.

No untoward circumstance seems to have followed the occlusion of the carotids in this case. In a letter from Prof. Park received a fortnight following the operation, he states that the patient is free from pain and would soon return home. He had a rather sharp secondary hemorrhage from the scalp on the afternoon of the operation, but this was easily controlled. A few days later he had a discharge from the opposite ear, which soon subsided. At the time of writing he had a purulent discharge from the left nostril, probably, as stated by Prof. Park, from the antrum.

It is difficult to trace any pathological connection between the operation and these post-operative complications. This relates to both the intracranial neurectomy and the provisional occlusion of the carotids. It is barely possible that the circulatory disturbances incident to the latter may account for them, although just in what manner is not at the time of writing at all clear.

301 DeKalb Avenue.

